# Chemical Analysis and Investigation of Biological Effects of *Salvia officinalis* Essential Oils at Three Phenological Stages

**DOI:** 10.3390/molecules27165157

**Published:** 2022-08-12

**Authors:** Hamza M. Assaggaf, Hanae Naceiri Mrabti, Bodour S. Rajab, Ammar A. Attar, Reema A. Alyamani, Munerah Hamed, Nasreddine El Omari, Naoual El Menyiy, Zakaria Hazzoumi, Taoufiq Benali, Samiah Hamad Al-Mijalli, Gokhan Zengin, Yusra AlDhaheri, Ali H. Eid, Abdelhakim Bouyahya

**Affiliations:** 1 Department of Laboratory Medicine, Faculty of Applied Medical Sciences, Umm Al-Qura University, Makkah 21955, Saudi Arabia; 2 Laboratory of Pharmacology and Toxicology, Bio Pharmaceutical and Toxicological Analysis Research Team, Faculty of Medicine and Pharmacy, Mohammed V University in Rabat, Rabat 10100, Morocco; 3 Clinical Nutrition Department, Faculty of Applied Medical Sciences, Umm Al-Qura University, Makkah 24381, Saudi Arabia; 4 Department of Pathology, Faculty of Medicine, Umm Al-Qura University, Makkah 21955, Saudi Arabia; 5 Laboratory of Histology, Embryology, and Cytogenetic, Faculty of Medicine and Pharmacy, Mohammed V University in Rabat, Rabat 10100, Morocco; 6 Laboratory of Pharmacology, National Agency of Medicinal and Aromatic Plants, Taouanate 34025, Morocco; 7 Plant and Microbial Biotechnology Center-Moroccan Foundation for Advanced Science, Innovation and Research, Rabat 10100, Morocco; 8 Environment and Health Team, Polydisciplinary Faculty of Safi, Cadi Ayyad University, Safi 46000, Morocco; 9 Department of Biology, College of Sciences, Princess Nourah Bint Abdulrahman University, Riyadh 11671, Saudi Arabia; 10 Department of Biology, Science Faculty, Selcuk University, Konya 42130, Turkey; 11 Department of Biology, College of Science, United Arab Emirates University, Al Ain 15551, United Arab Emirates; 12 Department of Basic Medical Sciences, College of Medicine, QU Health, Qatar University, Doha 2713, Qatar; 13 Laboratory of Human Pathologies, Faculty of Sciences, Mohammed V University in Rabat, Rabat 10106, Morocco

**Keywords:** *Salvia officinalis*, essential oils, phenological stages, anti-inflammatory effects, antidiabetic activity

## Abstract

*Salvia officinalis* is a medicinal plant used to treat some diseases, including microbial infections and diabetes. Different studies showed the biological and pharmacological properties of this species. The aim of this study was the determination of the chemical compounds of *S. officinalis* essential oils and the investigation of their antimicrobial, antioxidant, antidiabetic, and anti-inflammatory properties. The chemical compounds of *S. officinalis* were determined by GC-MS analysis. The antioxidant activity was assessed by DPPH, ABTS, H_2_O_2_, and FRAP assays. The in vitro antidiabetic effect was evaluated by the inhibition of α-amylase, α-glucosidase, and lipase activities, and the anti-inflammatory effect was evaluated using the 5-lipoxygenase assay. Moreover, antibacterial activity was assessed against six bacterial strains using agar well diffusion assay and microdilution method. The main compounds in essential oils of *S. officinalis* at three phenological stages were naphthalenone, camphor, 1.8-cineole, and α-thujone. The full flowering stage essential oil showed the best antioxidant activity with different IC_50_ values according to the used tests. This oil also exhibited important inhibitory effects at the full flowering stage against α-amylase (IC_50_ = 69.23 ± 0.1 μg/mL), α-glucosidase (IC_50_ = 22.24 ± 0.07 μg/mL), and lipase (IC_50_ = 37.3 ± 0.03 μg/mL). The 5-lipoxygenase inhibitory effect was the best at the full flowering stage (IC_50_ = 9.24 ± 0.03 μg/mL). The results of the antibacterial evaluation revealed that, at three seasonal periods, *S. officinalis* essential oil demonstrated strong antibacterial activity. Although the full flowering stage had the best antibacterial activity, there were no significant differences between the three stages. Additionally, the essential oils showed bactericidal effects on *Listeria monocytogenes*, *Staphylococcus aureus*, *Bacillus subtilis*, *Proteus mirabilis*, *Escherichia coli*, and *Salmonella typhimurium*, respectively. The findings of this work showed remarkably that *S. officinalis* synthesizes essential oils according to different developmental stages. Moreover, it has exhibited interesting biological and pharmacological properties justifying its medicinal effects and suggesting it as a very important source of natural drugs.

## 1. Introduction

Medicinal plants are taken a great interest as source of bioactive compounds used for the treatment of different human pathologies [1,2,3].

The species of the genus *Salvia* constitute the largest genus of the Lamiaceae family [4]. Plants belonging to this genus are distributed in the South-East Asia, Central America, and the Mediterranean region [5]. Many species and their essential oils are reputed for their biological activities [6,7,8] and are commonly used in drugs, food, perfumery, and cosmetics [9]. There are several studies on the phytochemical characterization of a large number of *Salvia* species, which show the presence of numerous bioactive molecules, especially essential oils and phenolic compounds [5,10,11,12,13,14,15].

Recent studies have confirmed that various factors such as the part of the plant used, the ecological conditions, the harvesting season, and the growth stage, play an important role in the growth of medicinal plants and their content of active compounds in medicinal plants [4,13,16]. For these reasons, finding the optimal conditions to obtain the highest content of bioactive compounds in essential oils (EO) is a challenge for scientific researchers. In recent years, research has intensified to determine the correlation between environmental parameters, in particular phenological periods, the content of bioactive compounds in essential oils and their biological effectiveness. In this context, several studies have been carried out on species of the *Salvia* genus. Indeed, Saffariha and colleagues [16] demonstrated that the vegetative stage of *Salvia limbata* is the optimal harvest time to extract the highest content of *α*-pinene and *β*-pinene. Similarly, Farhat and collaborators [17] showed that the highest yield of *Salvia officinalis* essential oil from Tunisia was at the flowering phase. For the chemical analysis, the flowering stage was characterized by the highest 1,8-cineole content. The viridiflorol and camphor content evolved during the growth cycle. Another research work investigated the variation in the quantity and quality of the essential oil of *S. officinalis* during its life cycle stages. The results of this study demonstrated that the best yield of essential oil was obtained in the floral budding stage. Moreover, phytochemical analysis revealed that oxygenated monoterpenes were the main compounds at the fruiting with important concentrations [18].

Sage (*S. officinalis* L.) is a fragrant and medicinal herb well known for its pharmacological characteristics. It is a woody-stemmed perennial evergreen subshrub with grey leaves and blue to violet blooms. It is a member of the Lamiaceae family and is indigenous to the Mediterranean region, but it grows in other places around the world [19]. *S. officinalis* has traditionally been used in ethnomedicine to treat a variety of conditions, including gout, seizures, rheumatism, ulcers, hyperglycemia, disorientation, inflammation, tremor, diarrhea, and paralysis, and this plant has been the subject of many studies in recent years to verify its traditional use and discover novel biological effects. These assays have indicated a wide range of pharmacological actions, including hypolipidemic, anti-inflammatory, antioxidant, antinociceptive, antimutagenic, antibacterial, hypoglycemic, anti-dementia, and anticancer properties [20,21,22,23]. As is known, essential oils extracted from plants collected in different regions of the world in different seasons include different chemical molecules and may have different biological effects [24].

To the best of our knowledge, no report on the variation of essential oil composition, or biological effects of *S. officinalis* essential oils (SOEO) collected, at three phenological stages, from Morocco, is available. Therefore, the aim of this work was to find the optimal phenological stage to harvest this medicinal plant for its use in traditional medicine or for possible use in the pharmaceutical industries, as well as to evaluate the in vitro antibacterial, antioxidant, and antidiabetic effects of SOEO at three phonological stages: vegetative, beginning flowering, and full flowering.

## 2. Results

### 2.1. Chemical Composition

In order to properly assess the chemical variation of SOEO, it was important to study this during its various phases of seasonal development. Indeed, the study of the chemical composition of plant species according to different phenological phases constitutes a tool for monitoring phytochemical variation with respect to climate changes.

**Figure 1:**
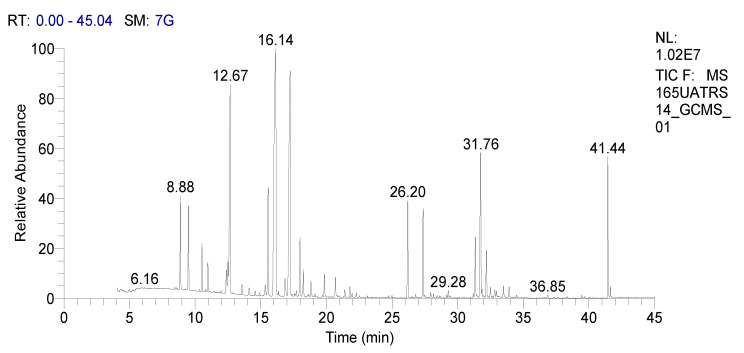
GC chromatography of SOEO at vegetative stage.

**Figure 2:**
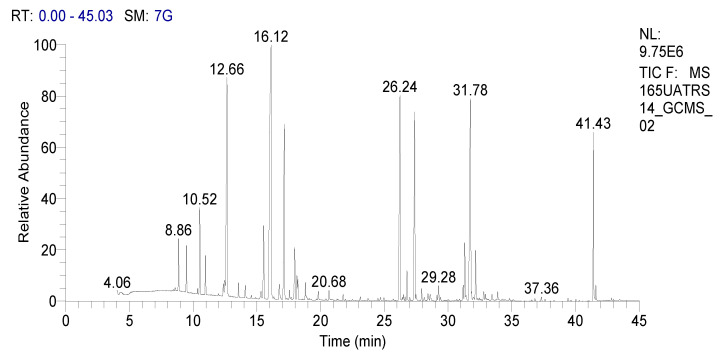
GC chromatography of SOEO at the beginning of the flowering stage.

**Figure 3:**
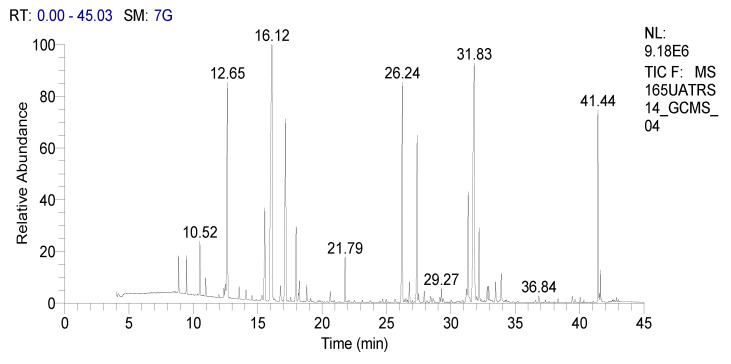
GC chromatography of SOEO at full flowering stage.

In our work we analyzed the chemical composition of SOEO during three growth stages: namely, the vegetative, the beginning flowering, and the full flowering stages using the GC-MS method (Figure 1, Figure 2 and Figure 3). A remarkable variation in the phytochemical composition depending on the development phase was observed. In total, forty-seven components were identified during certain stages while they were absent during others.

Regarding oxygenated monoterpenes, naphthalenone was the major compound with percentages of 22.9, 22.39, and 20.81% for the vegetative stage (VS), beginning of flowering stage (BFS), and full flowering stage (FFS), respectively. The second main compound was camphor, which showed the highest percentage during the vegetative stage (16.29%), followed by 1.8-cineole (12.51%), and α-thujone (4.46%). However, for sesquiterpene hydrocarbons, the full flowering stage showed the highest concentrations of major compounds; trans-caryophyllene (9.61%), eremophilene (8.37%), and α-humulene (8.34%) (Table 1).

Concerning the hydrocarbon monoterpenes, camphene and α-pinene showed the best concentrations. However, sage EO demonstrated low levels of oxygenated sesquiterpenes (ledeneoxide, (−)-caryophyllene oxide, and aromadendrene oxide).

### 2.2. Antibacterial Effect

**Figure 4:**
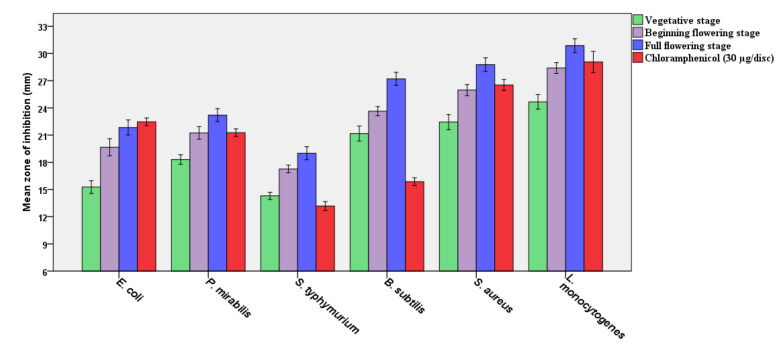
Mean zone of inhibitions of the three developmental stages compared to chloramphenicol.

The in vitro antimicrobial activities of SOEO at three developmental stages were performed using the disc diffusion technique against six bacterial strains (Figure 4). The results obtained are expressed as the mean zone of inhibitions of the three developmental stages compared to chloramphenicol and were highly significant as being antibacterial against all tested bacteria when compared to this antibiotic (*p ≤* 0.05), taking into account that the EO was in pure form and the chloramphenicol was at a concentration of 30 µg/disc. The results revealed no significant variations in antibacterial activity between the EO of this plant’s three development phases, reflecting the stability of the bioactivity of the EO of the plant during these three phonological stages. *Listeria monocytogenes* had the highest inhibitory zone, followed by *Bacillus subtilis*, *Staphylococcus aureus*, *Proteus mirabilis*, *Escherichia coli*, and *Salmonella typhimurium* (Table 2). Although no significant changes were found between the three development phases, the full flowering stage demonstrated stronger antibacterial activity than the other stages.

The minimum inhibitory concentration (MIC) and the minimum bactericidal concentration (MBC) tests were performed to reveal the possible bactericidal and/or bacteriostatic effects of SOEO, and the results are recorded in Figure 4, with the lowest MIC and MBC values indicating the highest inhibitory effect; if MBC values were less than or equal to four, they were considered bactericidal, and if higher than four, they were considered bacteriostatic, and according to the MBC results, the EO has a bactericidal effect. Interestingly, the findings confirm the disc diffusion assay, with the lowest MIC and MBC values recorded with *L. monocytogenes*, followed by *S. aureus*, *B. subtilis, P. mirabilis,* and *E. coli,* and the highest MIC and MBC values reported with *S. typhimurium*.

### 2.3. Antioxidant Activity

Medicinal plants are reserves of natural bioactive substances possessing several antioxidant properties in different biological systems. In the present study, the antioxidant effect of SOEO harvested at three developmental stages was evaluated using three assays: DPPH, FRAP, and ABTS assays. The results are shown in Table 3 and the IC_50_ values were calculated to compare these results to those of Trolox and ascorbic acid, which were used as reference standards. According to the recorded results, SOEO exhibited good antioxidant activities in the three tests but these were relatively lower than the standard antioxidants ascorbic acid and Trolox. The DPPH, FRAP, and ABTS tests demonstrated the best antioxidant capacity of SOEO at the full flowering stage with IC_50_ values of 113.56 ± 3.29, 126.85 ± 2.17 and 141.55 ± 1.81 μg/mL respectively. The SOEO at vegetative stage presented the lowest activity, with IC_50_ values of 188.43 ± 2.46, 212.91 ± 3.88, and 244.65 ± 1.74 μg/mL respectively. The Trolox and ascorbic acid values for the three tests were 28.19 ± 1.12 and 17.73 ± 0.74 μg/mL for DPPH, 69.55 ± 1.75 and 42.91 ± 1.17 μg/mL for FRAP, and 71.48 ± 1.72 and 56.84 ± 2.05 μg/mL for ABTS, respectively. The difference observed in the antioxidant potential of the SOEO from different phenological stages is a consequence of variations in the concentrations of individual bioactive compounds, which can be modulated by the impact of environmental growing conditions.

### 2.4. Antidiabetic Activity

The management of metabolic disorders requires the blockage of carbohydrates hydrolyzing enzymes as the first strategy of prevention and treatment. α-Amylase, α-glucosidase and pancreatic lipase are important digestive enzymes implicated in the intestinal metabolism of lipids and carbohydrates. Therefore, targeting and suppressing these enzymes is an effective means to disrupt glucose absorption and prevent postprandial increases of glycaemia levels, which may probably avoid diabetes progression. In this regard, the effect on α-amylase, α-glucosidase, and lipase of SOEO at three developmental stages was evaluated. The results are listed in Table 4 and compared to those of Acarbose and Orlistat, used as positive controls. SOEO at three phenological stages exerted important inhibitions of enzymes tested. Furthermore, for the full flowering stage, a significant inhibition was observed after SOEO treatment against α-amylase, α-glycosidase, and lipase compared to the other two stages, with IC_50_ values of 69.23 ± 0.1, 22.24 ± 0.07, and 37.3 ± 0.03 μg/mL, respectively. However, its activity remained lower when compared to Acarbose’s and Orlistat’s effects, which have potent inhibitory effects on the activity of both enzymes. In addition, the effect of EO at vegetative stage was weaker, with an IC_50_ of 121.54 ± 0.02, 59.11 ± 0.03 and 83.47 ± 0.11 µg/mL for α-amylase, α-glycosidase, and lipase, respectively.

### 2.5. Anti-Inflammatory Effects

Lipoxygenase (LOX) is a family of lipid-peroxidizing enzymes that catalyze the peroxidation of arachidonic acid. This product plays crucial roles in inflammatory processes and is implicated in various pathogenesis. Therefore, the use of a molecule that suppresses lipoxygenase activity could be of primary importance in the treatment of inflammatory-related diseases. Many studies have evaluated natural products as possible anti-inflammatory agents. However, the present work studied the anti-inflammatory effect of SOEO at three developmental stages by testing its ability to inhibit the activity of lipoxygenase (Table 5). The results showed that SOEO at the full flowering stage exhibits the best inhibitory activity with IC_50_ value of 9.24 ± 0.03 µg/mL. However, when compared to quercetin (IC_50_ = 4.89 ± 0.02 µg/mL), the tested sample showed a lower anti-inflammatory effect. In addition, the effect of EO at vegetative stage was weaker, with IC_50_ value of 54.39 ± 0.01 µg/mL.

## 3. Discussion

In this work, the chemical composition and biological effects of SOEO at three phenological stages were investigated. Results of phytochemical analyses showed that SOEO contains different volatile compounds with some variability between the three phenological stages. Some molecules detected in our study were also found by Ahmadi and Mirza, [25] and Baranauskiene et al. [26] who used the same analytical method (GC-MS) in the evaluation of SOEO chemical composition at different growth stages. Moreover, our findings were in line with those described by Hossein Mirjalili et al. [18] by revealing the predominance of the group of oxygenated monoterpenes in sage EO during its phenological cycle. However, Ben Farhat and collaborators [17] revealed that 1,8-cineole is the major compound of this plant with percentages varying according to the phenological period (17.64–20.44%) [17]. Taking into account this link between phytochemical variations and seasonal climate variations, the origin of the species will also be an important factor in this relationship [27]. Indeed, Chalchat et al. [28] were among the first authors to confirm this evidence. They selected species of five clones of different origins, namely, the Czech Republic, Romania, Portugal, Hungary, and France, where they recorded a variation of certain molecules according to the origin. Interestingly, SOEO collected from Algeria presented a percentage of 1,8-cineole (12.30%) [29] and camphor (16.86%) [30] that was very similar to that of our study (12.51 and 16.29%, respectively). Moreover, *S. officinalis* grown in different regions (Tunisia, Brasil, Spain, etc.) showed a high diversity in the main, components being identical to those found in our oil, analyzed by GC-FID and/or GC-MS or by their combination [31,32,33,34]. On the other hand, several investigations have revealed the involvement of other exogenous factors in the quality as well as the quantity of SOEO, such as the drying method used [35], the applied environmental conditions [36], the supplemented compounds [37], and the chosen hydrodistillation time [38].

Furthermore, SOEO exhibited significant antibacterial activity during three seasonal periods. Although the full flowering stage has the highest antibacterial activity, the three phases have no significant differences. Additionally, EO has been shown to be bactericidal against *S. aureus, L. monocytogenes*, *B. subtilis, P. mirabilis, E. coli*, and *S. typhimurium*. Indeed, determining the inhibitory zone is a qualitative procedure. The MIC and MBC values were calculated in this respect, since they provide information regarding the bactericidal and bacteriostatic effects of these oils. Concentrating on MIC and MBC presently, the full-flowering stage had the strongest bactericidal activity versus the bacteria tested, compared to the vegetative and beginning-flowering phases, although these variations were statistically non-significant (*p* ≤ 0.05). Previous studies support out findings and claim that the effective antibacterial activity is attributed to the major phytochemical molecules of SOEO, which are α-thujone, 1,8-cineole, *β*-pinene, borneol, and camphor [39]. Additionally, similar to our results, SOEO extracted in various countries had substantial antibacterial action against many Gram-negative and Gram-positive bacteria [40,41,42]. Moreover, our investigation substantiates the previously published presumption that SOEO has a bactericidal effect against various bacterial strains [43,44]. As can be observed, the growth of bacteria tested was affected differentially by the components of SOEO, indicating that various components may have distinct modes of action or that certain bacteria’s metabolisms are more capable of overcoming or adapting to the oil’s influence [45]. Additionally, our results concur with the assumption that Gram-positive bacteria are more vulnerable to EOs than Gram-negative bacteria [46,47,48].

Moreover, certain compounds have an inhibitory oxidation power [49,50]. Therefore, this interesting antioxidant effect of our oils can be attributed to the significantly higher concentration of oxygenated monoterpenes, including naphthalenone, camphor, 1.8-cineole and α-thujone, as well as sesquiterpenes such as eremophilene, α-humulene and trans-caryophyllene, or to the synergistic effects of its compounds. Our findings are in agreement with our previous studies on the antioxidant activity of SOEO [33,51], which have confirmed the potential role of EOs as natural antioxidants and in the preventive effect, which aims to prevent the appearance of several pathologies caused by reactive oxygen species such as cancer, diabetes, and cardiovascular diseases [52,53].

Concerning in vitro antidiabetic effects, our results are in agreement with those obtained by Mahdi et al. [54] who examined the α-glucosidase and α-amylase inhibitory activities of *S. officinalis* methanol extract and fractions of ethyl acetate and n-butanol in vitro. The authors demonstrated that the ethyl acetate fraction exhibits the best anti-diabetic activity, with IC_50_ values of 46.52 ± 2.68 and 104.58 ± 0.06 mg/mL respectively. Additionally, Pereira et al. [55] revealed important anti-diabetic activities (IC_50_ = 71.2 ± 5 and 4.6 ± 3.6 µg/mL for α-glucosidase and lipase enzymes) with *S. officinalis* aqueous extract. The anti-lipase activity of *S. officinalis* methanolic extract of the leaves has been also described by Ramirez et al. [56], who reported an EC_50_ value of 94 µg/mL. On the other hand, other in vivo studies have targeted the hypoglycemia activity of this plant using different extracts and experimental models. Eidi and collaborators [57] revealed that the administration of *S. officinalis* alcoholic extract (0.1, 0.2, and 0.4 g/kg body weight) significantly reduced blood glucose levels and increased plasma insulin in streptozotocin (STZ)-induced diabetic rats. Similarly, Alarcon-Aguilar et al. [58] investigated this effect by administering a water–ethanolic extract to alloxan-induced diabetic rats and showed a decrease in blood glucose levels compared to glibenclamide, which is used as an antidiabetic drug. Using the same animal model, the same results were recorded by Baricevic et al., [59] who demonstrated that essential oil administration significantly reduces blood glucose levels in diabetic animals. These activities are certainly due to the main volatile compounds of the plant, which are known for their antidiabetic potential, including camphor, 1.8-cineole, α-thujone, trans-caryophyllene, and naphthalenone. Indeed, Kuranov et al. [60] studied the antidiabetic potential of camphor in vivo and in vitro using the OGTT in male albino mice and dipeptidyl peptidase-4 (DPP-4) inhibition, respectively. They showed that this molecule strongly inhibits the activity of DPP-4 with IC_50_ values varying between 1.27 and 15.78 μM and it reduces fasting glycaemia. Likewise, 1.8-cineole showed a potent inhibitory power against α-amylase (IC_50_ = 0.78 ± 0.05 mg/mL) [61]. Moreover, oral administration of α-thujone (60 mg/kg/day) decreases the plasma glucose level in STZ-Induced diabetic rats, which correlated with increased glycogen production via the activation of the Akt/GSK-3β signaling pathway [62]. Concerning trans-caryophyllene, Suijun et al. [63] and Basha and Sankaranarayanan, [64] showed that this compound is able to restore certain parameters related to diabetes, including the regulation of glucose-stimulated insulin secretion (GSIS) in pancreatic *β*-cells via activation of the cannabinoid type 2 receptor (CB_2_R), protecting pancreatic *β*-cells, decreasing the glycaemia, and increasing plasma insulin levels. Furthermore, bioactive substances capable of inhibiting digestive enzymes and at the same time possessing potential antioxidant activities could be used to control blood glucose levels and the oxidative stress accompanying diabetes [65].

Concerning the anti-inflammatory effects, our data are similar to previous studies demonstrating the anti-inflammatory activity of SOEO collected from different countries. For instance, El Euch et al. [32] revealed that the Tunisian SOEO was able to inhibit the 5-lipoxygenase enzyme with an IC_50_ value of 36.15 ± 1.27 mg/L. Albano et al. [66] demonstrated also that the oil of *S. officinalis* collected from Portugal significantly inhibited this enzyme with an IC_50_ value 827.9 ± 60.6 mg/L. Moreover, Chehade et al. [67] studied the anti-inflammatory potential of Lebanese SOEO using the albumin denaturation inhibition assay and revealed that EO significantly decreased dose-dependent albumin denaturation. Furthermore, Brazilian *S. officinalis* oil significantly inhibited in vitro leukocyte chemotaxis caused by casein and reduced, in vivo, the amount of adhesion, rolling, and leukocytes’ migration in carrageenan-induced inflammatory [68]. The anti-inflammatory activity of SOEO may be attributed to its constituents that have been demonstrated to be involved in inflammatory treatments, namely 1.8-cineole (10.75%), naphthalenone (20.81%), camphor (14.35%), α-thujone (2.94%), trans-caryophyllene (9.61%), α-humulene (8.34%) and eremophilene (8.37%). An in vivo test conducted by Chen et al. [69] revealed that 1,8-cineole exerted an important anti-inflammatory effect in a trinitrobenzene sulfonic acid (TNBS)-induced colitis model in rats via the inhibition of myeloperoxidase production. Moreover, it was shown that treatment with camphor significantly reduced paw volume in a turpentine-induced animal model of inflammation [70]. The study of Ehrnhöfer-Ressler and their colleagues demonstrated that treatment of the human gingival fibroblasts with either camphor, 1,8-cineole, and thujone reduces the release of proinflammatory interleukins with a mean percentage of inhibition of 67−76 and 50−61% for phorbol-12-myristate-13-acetate/ionomycin-stimulated IL-8 and IL-6 secretion [71].

## 4. Materials and Methods

### 4.1. Reagents

Acarbose, quercetin, ascorbic acid, 2,2′-diphenyl-1-picrylhydrazyl (DPPH), 2,2-azino-bis-3-ethylbenzothiazoline-6-sulfonic acid (ABTS), 6-hydroxy-2,5,7,8-tetramethylchroman-2-carboxylic acid (Trolox), α-amylase, and α-glucosidase, nystatin, and ascorbic acid were purchased from Sigma-Aldrich (Saint-Quentin-Fallavier, France). Lipoxygenase (5-LOX) and linolenic acid were purchased from Sigma-Aldrich (Saint-Louis, MO, USA). Mueller–Hinton Agar, DMSO, and chloramphenicol were purchased from (Biokar, Beauvais, France). All other reagents were of analytical grade.

### 4.2. Plant Collection and Extraction

In this study, aerial parts (flowering tops) of *S. officinalis* were harvested from Boutaher (34°29′52.3″ N 4°47′11.9″ W) in the region of Taounate, Northwest of Morocco. The plant was harvested in three phenological stages of the plants: the vegetative stage (May); beginning of the flowering (June) stage; full flowering stage (July). The samples were air dried at room temperature in the shade. Then, an amount of 100 g of dried flowering tops (a mixture of leaves and flowers) of *S. officinalis* was subjected to hydrodistillation for three hours using the Clevenger type device. Each extraction assay has been performed with three replicates and the recovered EO was separated from the aqueous phase using a separating funnel. The EO thus obtained was dehydrated with anhydrous sodium sulphate, weighed then stored at 4 °C until use in the upcoming experiments.

### 4.3. GC-MS Analysis of Essential Oils

The chemical components of SOEO were determined using gas-chromatography/mass-spectrometry (GC/MS) analysis as described previously. Indeed, a Hewlett-Packard (HP6890) GC instrument coupled with a HP5973 MS and equipped with a 5% phenylmethyl silicone HP-5MS capillary column (30 m × 0.25 mm × film thickness 0.25 μm) was used in GC analysis. The used column temperature increased from 50 °C for 5 min to 200 °C with a 4 °C/min rate. Helium with a 1.5 mL/min flow rate and split mode (flow: 112 mL/min, ratio: 1/74.7) was the used carrier gas. The hold time was 48 min, while the temperature of the injector and detector was 250 °C.

The machine was led by a computer system type ″HP ChemStation″, managing the functioning of the machine and allowing us to follow the evolution of chromatographic analyses. Diluted samples (1/20 in methanol) of 1 μL were injected manually. In addition, 70 eV ionization voltage, 230 °C ion source temperature, and 35–450 (*m*/*z*) scanning range were the MS operating conditions. Finally, the qualitative quantification of the different compounds was based on the percentage area of each peak of the sample compounds and was confirmed by reference to their MS identities (Library of NIST/EPA/NIH MASS SPECTRAL LIBRARY Version 2.0, build 1 July 2002).

### 4.4. Antibacterial Activity

#### 4.4.1. Bacterial Strains

The antibacterial activity was evaluated against the following six bacterial strains representing Gram-positive and Gram-negative bacteria: *E. coli* ATCC 25922, *P. mirabilis* ATCC 25933, *S. typhimurium* ATCC 700408, *B. subtilis* ATCC 6633, *S. aureus* ATCC 29213, and *L. monocytogenes* ATCC 13932.

#### 4.4.2. Disc Diffusion Assay

The primary screening of the antimicrobial activity of the studied samples was evaluated by the disc diffusion method according to the previous published [72]. Briefly, the culture suspension of each species was inoculated in the optimal culture medium (Mueller-Hinton Agar for bacteria, and Sabouraud agar for yeast and fungi). Afterwards, 6 mm diameter sterile paper discs soaked with 10 µL of SOEO (mixed with 5% of DMSO) of the three phenological stages were deposited on each plate. Chloramphenicol (30 µg) was used as a positive control for bacteria and nystatin (100 I.U.) was used as a positive control for yeast and fungi, while DMSO (10 µL; 5%) was used as a negative control. The plates were incubated at the following growth conditions; 37 °C for 24 h, 25 °C for 48 h, and 25 °C for 72 h, for bacteria, yeast and fungi, respectively. After incubation, the inhibitory diameters were measured in millimeters and the results are expressed as means ± standard deviation of three replicates.

#### 4.4.3. Determination of MIC and MBC

The broth microdilution experiment was employed to determine the MIC as previously reported [73]. The MBC corresponds to the minimum concentration of sample that can kill the microorganism. The same microdilution experiment derived from the determination of MIC was used. After the incubation, 10 μL of each tube that did not present visible growth was sub-cultured on Tryptone Soy Agar (Biokar, Beauvais, France) and incubated at 37 °C for 24 h, and the lowest concentration that did not present any growth on media was considered as the MBC [74].

### 4.5. Antioxidant Activity

The antioxidant activity of SOEO was evaluated by using four complementary spectrophotometric methods, DPPH and ABTS (transfer of both a hydrogen atom and an electron), FRAP (the transfer of an electron), and H_2_O_2_-scavenging assays, according to the previous published protocols [75,76,77]. The results are expressed as the concentration of essential oils providing 50% inhibition (IC_50_) and were calculated by plotting the inhibition degrees against the essential oils concentrations. Trolox and ascorbic acid were used as positive controls. The assays were carried out in triplicate and IC_50_ values were presented as means ± SD.

### 4.6. In Vitro Anti-Diabetic Assay

The anti-diabetic effect of SOEO was determined by testing the ability of the oils to inhibit the enzymatic effect of α-amylase and α-glucosidase according to the same methods as our previous published study [78] and the determination of lipase inhibitory activity was according to the method described by Hu et al. [79]. For the test of α-amylase, a volume of 250 μL of EOs and 250 μL of 0.02 M sodium phosphate buffer (pH = 6.9) containing α-amylase at 240 U/mL was incubated for 20 min at 37 °C. Then, 250 μL of 1% starch solution prepared in 0.02 M sodium phosphate buffer (pH = 6.9) was added, followed by incubation for 15 min at 37 °C. Then, 1 mL of DNS was added, and incubation of the mixture in a boiling water bath for 10 min was carried out. The reaction mixture was diluted by adding 2  mL of distilled water, and absorbance was measured at 540 nm with a spectrophotometer. In this rest, acarbose was used as a positive control. For the test of α-glucosidase, 200 μL of EOs and 100 μL of 0.1 M sodium phosphate buffer (pH = 6.7) containing the enzyme α-glucosidase solution (0.1 U/mL) was incubated at 37 °C for 10 min. After preincubation, 200 μL of 1 mM pNPG solution in 0.1 M sodium phosphate buffer (pH = 6.7) was added. After incubation at 37 °C for 30 min, 1 mL of 0.1 M of Na_2_CO_3_ was added, and the absorbance was recorded at 405 nm. The α-amylase and α-glucosidase inhibitory effects are expressed as percentage inhibition, and the IC_50_ values were determined.

### 4.7. Lipoxygenase (5-LOX) Inhibition Assay

Lipoxygenase inhibitory activity of SOEO at three phenological stages was evaluated by following the linoleic acid oxidation at 234 nm, according to a previously published method [80]. Briefly, 20 µL of oil and 20 µL of 5-LOX from Glycine max (100 U/mL) were pre-incubated with 200 µL of phosphate buffer (0.1 M, pH 9), at room temperature for 5 min. The reaction was started by the addition of 20 µL of linolenic acid (4.18 mM in ethanol) and followed for 3 min at 234 nm. Results correspond to the mean ± SEM of three independent assays, each performed in triplicate. Quercetin was used as positive control.

### 4.8. Statistical Analysis

All experiments were conducted in triplicate and the obtained results are expressed as mean ± SD. Data were analyzed using SPSS Software (IBM SPSS statistics for Windows, Version 21.0. Armonk, NY, USA, IBM Corp) and comparisons between means were done using a one-way ANOVA, followed by the Tukey’s test. Differences between means were considered significant when *p* < 0.05.

## 5. Conclusions

Here, the chemical composition and some biological activities of SOEO were highlighted. It was revealed that this species expresses secondary metabolites deferentially according to each phenological stage. Moreover, biological activities including antidiabetic, antioxidant, and antibacterial effects showed different results with variability depending on the major bioactive compounds. These molecules constitute veritable drugs with antidiabetic, antioxidant and antibacterial activities. However, further investigations should be carried out to determine more the pharmacodynamic and pharmacokinetic parameters of these substances. Moreover, toxicological studies should also be conducted to validate their safety.

## Figures and Tables

**Table 1:** Chemical composition of *S. officinalis* at three phenological stages.

Peak Area%				
	RT	VS	BFS	FFS
Compounds	Monoterpenes			
*cis*-sabinene	10.52	0.9	0.97	1.24
*δ*.3-carene	13.57	1.43	0.83	1.13
*α*-pinene	14.11	3.2	2.26	2.66
naphthalene	24.50	0.23	0.15	0.2
*α*-terpinene	8.45	-	0.2	0.27
l-phellandrene	8.60	0.73	-	0.2
*β*-pinene	8.88	-	0.98	0.97
*ϒ*-terpinene	10.34	0.32	0.51	0.62
terpinolene	14.56	0.14	0.4	0.38
*cis*-ocimene	15.34	0.47	0.42	0.39
*β*-carene	16.21	0.55	0.95	0.36
camphene	9.49	2.74	1.65	1.87
azulene	28.45	0.35	-	-
Total		12.09	10.28	11
	**Oxygenated Monoterpenes**			
1.8-cineole	12.68	12.51	8.61	10.75
*α*-thujone	15.56	4.46	3.32	2.94
*p*-menthone	17.71	0.25	-	-
isoborneol	17.99	2.21	2.27	1.57
carveol	19.69	-	0.14	0.15
*Z*-citral	20.18	0.78	0.1	-
*β*-thujone	14.83	0.84	1.08	0.61
d-verbenone	19.12	0.13	0.11	-
*cis*-limonene oxide	20.80	0.75	0.24	0.3
naphthalenone	16.15	22.9	22.39	20.81
camphor	20.36	16.29	15.98	14.35
Total		61.12	54.24	51.48
	**Sesquiterpenes**			
*α*-bourbonene	24.99	0.12	0.13	-
trans-caryophyllene	26.20	3.66	8.91	9.61
germacrene D	25.12	0.15	0.14	0.16
aromadendrene	26.80	0.15	0.37	0.45
*α*-caryophyllene	31.86	0.2	0.14	0.14
*α*-humulene	27.80	3.36	7.09	8.34
Ledene	28.45	0.12	0.2	0.23
*cis*-Calamenene	29.41	0.12	0.1	0.11
eremophilene	28.63	7.25	7.4	8.37
cadinene	29.27	0.22	-	-
ë-cadinene	29.41	0.27	0.24	0.3
dehydroaromadendrene	30.93	0.14	0.24	0.21
junipene	32.51	-	-	-
valencene	26.64	0.11	0.11	0.13
ç-himachalene	30.71	0.53	0.23	0.22
Total		16.4	25.3	28.27
	**Oxygenated Sesquiterpenes**			
ledeneoxide	32.36	0.36	0.19	0.18
(−)-caryophyllene oxide	31.36	1.71	1.09	1.1
aromadendrene oxide	31.90	2.23	1.53	-
Total		4.3	2.81	1.28
	**Other Molecules**			
exobornyla cetate	21.78	0.34	2.14	1.24
sabinyla cetate	21.95	0.15	0.23	-
geraniol formate	10.96	0.33	0.34	0.36
myrtenyla cetate	23.13	-	0.22	-
linalyla cetate	32.02	0.18	-	-
**Total**		**1**	**2.93**	**1.6**

**VS**: Vegetative stage; **BFS**: beginning of the flowering stage; **FFS**: Full flowering stage.

**Table 2:** MIC and MBC of *Salvia officinalis* essential oils as percentages (*v*/*v*) at three developmental stages.

Microorganisms	Gram	*Salvia officinalis* Essential Oils in% (*v*/*v*)						Chloramphenicol (µg/mL)
		Vegetative Stage		Beginning Flowering Stage		Full Flowering Stage		
		MIC	MBC	MIC	MBC	MIC	MBC	
*E. coli* ATCC 25922	**Gram −**	1	2	0.5	1	0.5	0.5	4
*P. mirabilis* ATCC 25933	**Gram −**	1	1	0.5	1	0.5	0.5	4
*S. typhimurium* ATCC 700408	**Gram −**	2	2	1	2	1	1	64
*B. subtilis* ATCC 6633	**Gram +**	0.25	0.5	0.25	0.25	0.12	0.25	32
*S. aureus* ATCC 29213	**Gram +**	0.5	0.5	0.25	0.25	0.12	0.25	4
*L. monocytogenes* ATCC 13932	**Gram +**	0.25	0.5	0.12	0.25	0.12	0.12	2

**Table 3:** The antioxidant activity of SOEO (IC_50_ in μg/mL) at three developmental stages. The results are presented as means ± SD (standard deviations) for triplicate assays.

	Controls		**Essential Oils**		
	**Ascorbic Acid**	Trolox	Vegetative Stage	Beginning Flowering Stage	Full Flowering Stage
DPPH	17.73 ± 0.74 ^a^	28.19 ± 1.12 ^b^	188.43 ± 2.46 ^c^	149.19 ± 5.31 ^d^	113.56 ± 3.29 ^e^
FRAP	42.91 ± 1.17 ^a^	69.55 ± 1.75 ^b^	212.91 ± 3.88 ^c^	188.45 ± 3.17 ^d^	126.85 ± 2.17 ^e^
ABTS	56.84 ± 2.05 ^a^	71.48 ± 1.72 ^b^	244.65 ± 1.74 ^c^	198.05 ± 2.15 ^d^	141.55 ± 1.81 ^e^

Different letters indicate significant differences (*p* < 0.05; *n* = 3).

**Table 4:** The antidiabetic activity of SOEO (IC_50_ in μg/mL) at three developmental stages. The results are presented as means ± SD (standard deviations) for triplicate assays.

Assays	Essential Oils			Controls	
	Vegetative Stage	Beginning Flowering Stage	Full Flowering Stage	Acarbose	Orlistat
α-amylase IC_50_ (µg/mL)	121.54 ± 0.02 ^a^	81.91 ± 0.03 ^b^	69.23 ± 0.1 ^c^	40.71 ± 0.50 ^d^	-
α-glucosidase IC_50_ (µg/mL)	59.11 ± 0.03 ^a^	46.57 ± 0.01 ^b^	22.24 ± 0.07 ^c^	12.31 ± 0.05 ^d^	-
Lipase IC_50_ (µg/mL)	83.47 ± 0.11 ^a^	71.42 ± 1.13 ^b^	37.3 ± 0.03 ^c^	-	21.37 ± 0.05 ^d^

Different letters indicate significant differences (*p* < 0.05; *n* = 3), (-): not tested.

**Table 5:** The anti-inflammatory activity of SOEO (IC_50_ in μg/mL) at three developmental stages.

Assays	Essential Oils			Control
	Vegetative Stage	Beginning of the Flowering Stage	Full Flowering Stage	Quercetin
5-lipoxygenase	54.39 ± 0.01 ^a^	31.51 ± 0.02 ^b^	9.24 ± 0.03 ^c^	4.89 ± 0.02 ^d^

The results are presented as means ± SD (standard deviations) for triplicate assays. Different letters (a–d) indicate significant differences (*p* < 0.05; *n* = 3).

## Data Availability

Not applicable.
